# Tc-99m sulfur colloid SPECT-CT and assessment of functional liver reserve after Y90 radioembolization: A case report

**DOI:** 10.1016/j.ijscr.2019.08.009

**Published:** 2019-08-17

**Authors:** YeeMei Chan, WingHang Luk, LikFai Cheng, HoFung Chan, N.Y. Pan, KaFai Ma

**Affiliations:** Department of Diagnostic Radiology, LG1, Princess Margaret Hospital, Lai King Hill Road, Hong Kong

**Keywords:** AFP, alpha feto protein, BCLC, Barcelona Clinic Liver Cancer, CT, computed tomography, FRV, functional remnant volume, HCC, hepatocellular carcinoma, ICG-R-15, indocyanine green retention at 15 min, OSEM-3D, ordered 3-dimensional subset expectation maximization, SPECT, single photon emission computed tomography, Tc-99 MAA, Tc-99m macroaggregated albumin, Tc-99m SC, technetium-99m sulfur colloid, Y90, yttrium-90, Tc-99m sulphur colloid, SPECT -CT, Y90 radioembolization, Segmentectomy and lobectomy of liver

## Abstract

**Introduction:**

Quantitative assessment is an essential tool in determining the proportion of liver to be reserved before lobectomy. Technetium-99 m sulfur colloid single-photon emission computed tomography (Tc-99 m SC SPECT-CT) can help in the quantitative assessment of functioning liver tissues and percentage of liver reserve before segmentectomy and lobectomy Matesan et al. (2017), Bowen et al. (2016) and Lam et al. (2013).

**Presentation of case:**

A 64-year-old man with alcoholic cirrhosis was admitted to our hospital with a 15 × 10 x 13 cm bilobar HCC. Y90 radioembolization was utilized to downstage the liver tumor. On follow-up CT scan of the liver after radiotherapy, the HCC was much reduced to 6.5 cm in size but still viable with elevated alpha fetoprotein ([AFP] from 225 to 381 to 959 ng/mL). Resection was considered. Constitutional indocyanine green retention at 15 min (ICG-R-15) was 22%. We introduced the Tc-99 m SC SPECT-CT scan in order to assess the percentage liver function of each lobe. It showed minimal uptake in the remaining functioning right lobe with a hypertrophic left lobe to whole liver uptake ratio of 87.1%. This finding gave us confidence to perform right hepatectomy.

**Discussion:**

We used Tc-99 m SC SPECT-CT to estimate the normal functional liver reserve after Y90 radioembolization of a hepatocellular carcinoma (HCC). To our understanding, it is the first case report using Tc-99 m SC to predict the percentage of functional liver reserve after yttrium-90 (Y90) radioembolization.

**Conclusion:**

Tc-99 m SC SPECT-CT is a novel helper used to assess the differential liver function after Y90 radioembolization of HCC and before segmentectomy and lobectomy of the liver.

## Highlights

• Y90 radioembolization has shown feasibility for downstaging patients for resection.
• Functional liver reserve is a choice pre-surgery/postradioembolization measurement.
• Tc-99 m Sulfur Colloid SPECT-CT can assess functional liver reserve before surgery.

## 1 Introduction

Yttrium-90 (Y90) radioembolization is an effective treatment for unresectable hepatocellular carcinoma (HCC) that has shown feasibility as a tool for downstaging patients for resection [[4], [5], [6], [7], [8]]. However, postradioembolization, and prior to segmentectomy or lobectomy, it is important to assess the percentage of functioning liver reserve to better understand a patient’s prognosis and potential approaches to management. Tc-99 m sulfur colloid (Tc-99 m SC) is a diagnostic tracer that is taken up by reticuloendothelial cells of the liver and, when used in conjunction with single-photon emission computed tomography (SPECT-CT) and planar imaging, correlates with liver function in patients with HCC [1,2]. We specifically utilized this agent to assess normal functional liver reserve post-Y90 radioembolization and prior to surgery in a case where neither the patient nor the surgeon would have considered surgery as a feasible treatment option. The work has been reported in line with the SCARE criteria [9].

## 2 Case presentation

**Fig. 1:**
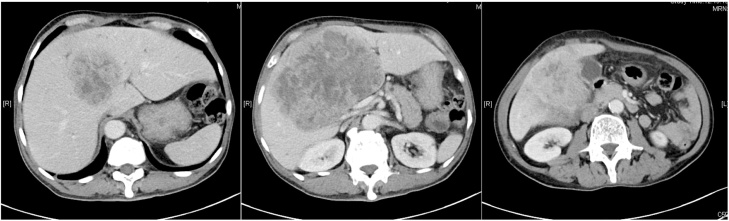
Axial image of CT scan before Y90 therapy: an HCC lesion 15 cm in size.

A 64-year-old man, referred by a private doctor, walked into the accident and emergency department of our major regional hospital. He had been experiencing abdominal discomfort for over a month, weight loss and poor appetite. He was a chronic smoker and user of alcohol, with benign colon polyps. He reported having no fever, tea-colored urine, or symptoms of gastrointestinal bleeding. Hard, irregular hepatomegaly was present on physical exam, and liver ultrasound revealed cirrhosis and bilateral liver masses. On admission, his liver function was within normal range. However, alpha feto protein (AFP) was 4522 ng/mL (Table 1). HbsAG was negative. An abdominal CT, performed one week later, showed a 15 × 10 x 13 cm bilobar mass, involving segments 4, 5, and 8 of the liver, without evidence of portal vein thrombus or extrahepatic spread. The patient was classified as having Barcelona Clinic Liver Cancer (BCLC) stage B disease, and a Child-Pugh score of A (Fig. 1). Hepatic angiogram revealed that approximately 90% of the tumor vasculature was supplied by right hepatic artery branches. Both portal veins were compressed by the tumor, but still patent.

**Table 1:** Record of laboratory results.

	Reference	Admission	6 Weeks After 1^st^ Admission	8 Weeks After 1^st^ Admission	10 Weeks After 1^st^ Admission | 1 Day Before Y90 Therapy	Y-90 Therapy	1 Month After Y90 Therapy	3 Months after Y90 Therapy	4 Months after Y90 Therapy	5 Months after Y90 Therapy	7 Months After Y90 Therapy | 1 Day Before Hepatectomy	Hepatectomy	7 Months After Y90 Therapy | 1 Day After Hepatectomy	7 Months After Y90 Therapy | 2 Days After Hepatectomy	7 Months After Y90 Therapy | 3 Days After Hepatectomy	7 Months After Y90 Therapy | 4 Days After Hepatectomy	7 Months After Y90 Therapy | 5 Days After Hepatectomy	7 Months After Y90 Therapy | 6 Days After Hepatectomy	7 Months After Y90 Therapy | 1 Week After Hepatectomy	8 Months After Y90 Therapy | 1 Month After Hepatectomy
AFP (ng/mL)	<9.1	4522	3386	6364	7425		225	381	959	573	na		na	na	na	na	na	na	na	14
Total bilirubin (umol/L)	5–21	11	9	10	12		7	12	11	12	11		19	15	15	16	12	11	9	9
ALP (U/L)	30–120	142	216	252	194		98	169	134	102	96		68	65	65	62	53	48	69	85
ALT (U/L)	<50	24	29	52	22		19	42	24	16	9		89	76	76	54	41	31	19	13
Albumin (g/L)	37–47	39	37	33	35		36	38	39	40	38		27	27	27	29	26	24	26	37
Globulin (g/L)	26–41	32	44	42	38		39	42	36	39	33		29	26	36	30	29	28	30	41
Hemoglobin, blood (g/dL)	13.4–17.1	13.0	9.4	8.9	7.7		na	na	12.8	na	12.5		10.5	9.6	10.6	10.0	9.8	12.5	10	na
WBC (× 10^9^/L)	3.7–9.2	5.7	7.1	6.2	6.4		na	na	6.5	na	6.3		11.8	8.1	5.8	5.0	4.7	6.3	9.5	na
Platelet (× 10^9^/L)	145–370	224	395	300	274		na	na	172	na	166		131	113	117	128	137	166	185	na
INR		1.0	1.1	1.0	1.1		1.0	1.0	1.0	1.0	1.1		1.1	na	1.1	1.1	1.0	1.1	1.0	na

AFP: alpha fetoprotein; ALP: alkaline phosphatase, total; ALT: alanine aminotransferase; INR: international normalized ratio; na: not available; WBC: white blood cell; Y90: yttrium-90.

Downstaging the tumor was a priority. Y90 radioembolization has been shown to contribute to a reduction in tumor size in patients with bulky disease, and was considered as a therapeutic choice. At 5 weeks post admission, Tc-99 m macroaggregated albumin (Tc-99 m MAA) SPECT-CT was utilized to assess the lung shunting percentage and tumor-to-normal (T/N) ratio [10], which were 9.06% and 1.56, respectively, and to determine the patient’s eligibility for Y90 radioembolization. In an attempt to promote a better T/N ratio, coil embolization of 2 branches of the right hepatic artery supplying S7 and S8, which did not supply the tumor, was conducted. Tc-99 m MAA SPECT-CT was repeated one week later at which time the T/N ratio improved to 3.1. Y90 radioembolization proceeded with administration of 3.7 GBq SIR-Spheres resin microspheres (SIRTEX Medical; Sydney, Australia): 0.92 GBq and 2.78 GBq to the left and right hepatic artery, respectively [11].

**Fig. 2:**
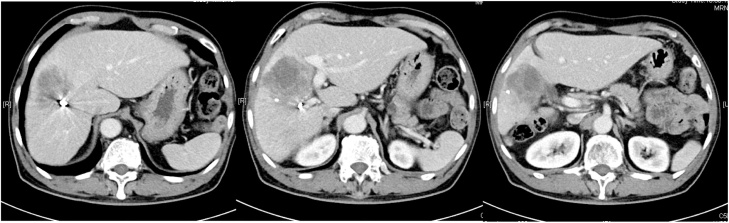
Axial images of CT scan after Y90 therapy: the HCC lesion reduced to 6.5 cm in size.

On follow-up CT scan of the liver 3 months after Y90 therapy, the tumor was reduced to 6.5 cm (Fig. 2). However, while the patient’s bilirubin levels remained within normal range, AFP levels began to rise, suggesting residual tumor activity (Table 1). At that time, resection was considered.

**Fig. 3:**
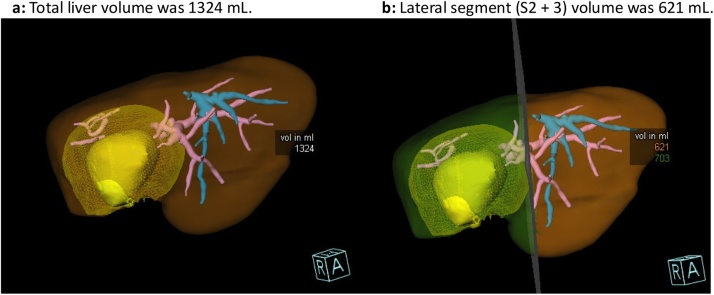
**a**,**b**: Liver volume was analyzed by the Syngo.Via (VB20A_HG05), Siemens Healthineers after Y90 therapy.

According to the follow-up CT scan, the volume of the lateral segment (S2 + S3) and the total liver volume were analyzed by Syngo.Via (Siemens Healthineers; Erlangen, Germany) at 621 mL and 1324 mL, respectively (Fig. 3a,b). The functional remnant volume (FRV) for right hepatectomy was 47% by volume (FRV% *= volume of reserve lobe / whole liver volume X 100%)*.

An indocyanine green (ICG) dye test, used to estimate hepatic functional reserve and ICG retention at 15 min, was 22%, higher than the common parameter for right hepatectomy, <14%. [12,13] After discussion with surgeons, oncologists, and radiologists, at five and a half months post Y90 radioembolization, a Tc-99 m SC SPECT-CT scan was introduced.

### 2.1 Tc-99 m sulfur colloid scintigraphy

#### 2.1.1 Procedure

An outside facility prepared 3 mCi of Tc-99 m SC, heated without filtrating [1], with the anticipation that these larger particles would accumulate mainly in the liver and spleen. The colloid was injected intravenously. Twenty minutes after injection, planar images (abdomen) and SPECT-CT with attenuation correction were performed. A dual-head SPECT-CT camera with low-energy, high-resolution collimators was used (GE Discovery NM/CT 670). A total of 60 pair views, each of 20-second duration, were acquired over 360°, in a 128 × 128 matrix. The SPECT-CT data were reconstructed using ordered 3-dimensional subset expectation maximization (OSEM-3D) with 2 iterations, 16 subsets, and a Butterworth filter (GE Xeleris Functional imaging workstation; GE Healthcare, Chicago, IL) [[1], [2], [3],^14^], The percentage uptake on the left lobe of total liver was calculated from both SPECT-CT data by OsiriX software (Geneva, Switzerland) and the 2 anterior and posterior planar views by using the geometrical mean method.

#### 2.1.2 Findings

**Fig. 4:**
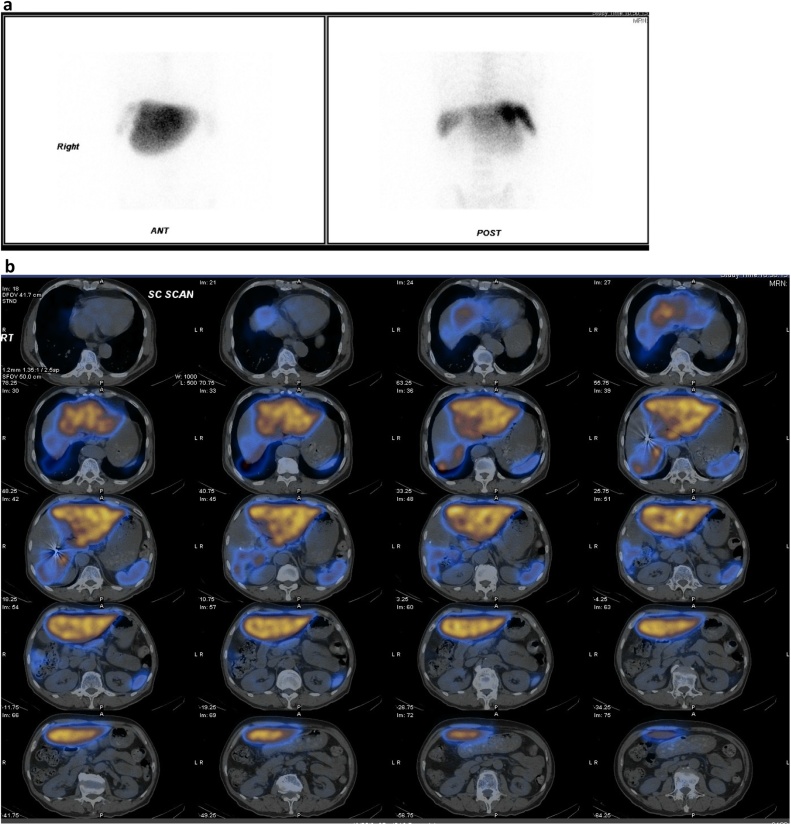
a) Planar view of abdomen in Tc-99 m colloid scintigraphy. Most of the colloid localized in the liver and spleen; only minimal uptake was found on the right lobe and a small amount on bone marrow. b) Fusion image of SPECT-CT in Tc-99 m sulfur colloid scintigraphy. The hypertrophic left lobe took up most of Tc-99 m SC injected; only minimal uptake was found on the right lobe.

A large photopenic area in the right lobe of the liver was observed, attributed to necrosis resulting from the Y90 treatment, with minimal uptake in the remaining areas of the right lobe. Conversely, the left lobe experienced liver hypertrophy (Fig. 4a,b). By using the falciform ligament as a boundary, the percentage of left lobe uptake to whole liver uptake was calculated at 87.1% *(% left lobe uptake = left lobe uptake count / total liver uptake count x 100%)*.

Liver function tests remained within the normal range (Table 1); ICG was 20% after recheck. As the tumor occupied a large volume of a portion of the liver that was determined to be nonfunctioning, extended right hepatectomy was offered to the patient, with consideration of both benefits and risks of the procedure.

During the operation, the right lobe and segment 4 of the liver were visualized. Both were shrunken and fibrotic, with the tumor at the right lobe encroaching onto the right pedicle. Viable tumor was also visualized at the gallbladder bed and segment 4/5. An enlarged lymph node was identified at the porta region. The patient’s albumin level was 26 g/L, in the week following surgery, however returned to 37 g/L one-month postsurgery (Table 1). No other complications were found.

Pathological analysis revealed a moderately differentiated HCC, stage pT3. The tumor was close to but not involving the capsular margin. The resection margin was negative for tumor, and the enlarged lymph node at the porta region did not contain metastatic tissue.

The patient was discharged on postoperative day 8 without complication and returned to the follow-up clinic one-month postoperation. He reported feeling well; had a fair appetite; no fever, wound, or other infection or disease; no weight loss or tea-colored urine; and walked unaided. The AFP was much reduced from 573 ng/mL to 14 ng/mL. Blood tests for liver function were normal demonstrating sustained functioning of the left liver lobe (Table 1).

## 3 Discussion

In this case study, a 15 × 10 x 13 cm HCC lesion was downstaged after Y90 radioembolization. However, the tumor remained viable prompting the consideration of lobectomy for this patient [15,16].

Liver volumetry by CT scan is commonly utilized prior to initiating liver segmentectomy or lobectomy. Yet, it can only offer a morphological volumetric assessment of the liver [17,18]. As seen in this patient, based on CT results, an FRV (47%) could not adequately reflect the physiological functioning of the remaining liver.

ICG-R-15 is an indicator of hepatic function that is often utilized prior to resection or transplantation [12]. However, it is difficult to determine functional liver reserve solely by ICG-R-15 as test results may be altered by regional hepatic blood flow and bilirubin levels [12]. In this patient, an ICG-R-15 of 22% caused the surgeons to reconsider the patient’s eligibility for surgery. Therefore, we introduced the Tc-99 m SC SPECT-CT scan as an alternative assessment tool.

Fusion images from the SPECT-CT system offered better localization and differentiation on the physiological uptake, with actual functional anatomy shown (Fig. 4a,b). The images were reconstructed using OSEM-3D with CT-based attenuation correction. As a result, the percentage of the segmental or lobar liver function to the whole liver function could be accurately calculated (*% of segmental or lobar liver function = segmental or lobar uptake count / the whole liver uptake count x 100%*).

In this case, the hypertrophic left lobe reported 87.1% of the whole liver function. A finding that encouraged our team to proceed with extensive right hepatectomy.

Although segmental liver function can be easily assessed by Tc-99 m SC SPECT-CT, motion artifacts must be carefully observed. Patient positioning and respiratory motion will cause misregistration on the resulting images. Advanced software algorithms can be used for motion correction in image co-registration of the abdomen and thorax in order to minimize artifacts from respiratory motion. Patient cooperation is also important, as it plays a key role in producing reliable and precise results, and can be aided by prior, clear physician-patient communication [19].

## 4 Conclusion

Tc-99 m SC SPECT-CT imaging may offer a physiological imaging method that overcomes the limitation of the morphological CT scan, as this simple, noninvasive procedure can evaluate segmental liver function. By combining information with ICG-R-15, Tc-99 m SC SPECT-CT imaging may serve as a useful tool in the planning of precise segmentectomy and an accurate preoperative predictor for functional liver reserve.

## Funding

We have no funding received for this study.

## Ethical approval

Ethnical approval is issued by the Kowloon West Cluster Research Ethics Committee (KWC-Rec) Hong Kong Hospital Authority.

## Consent

Written informed consent was obtained from patient for publication of this case report and accompanying images. A copy of the written consent is available for review by the Editor in-Chief of this journal on request.

## Author contribution

YeeMei Chan: Study concept, design, data collection, data analysis, interpretation and writing the paper.

WingHang Luk: Study concept, design, data collection, data analysis, interpretation and writing the paper.

LikFai Cheng: data interpretation and advisor.

HoFung Chan: data interpretation and advisor.

NY Pan: data interpretation and advisor.

KaFai Ma: data interpretation and advisor.

## Registration of research studies

Research Registry UIN: researchregistry4922.

## Guarantor

YeeMei Chan and WingHang Luk are assigned as the guarantors for this study.

## Provenance and peer review

Not commissioned, externally peer-reviewed.

## Declaration of Competing Interest

We have no conflicts of interest or financial ties to any person or organisation.
